# Newborn screening of primary carnitine deficiency: clinical and molecular genetic characteristics

**DOI:** 10.1186/s13052-025-01911-1

**Published:** 2025-03-11

**Authors:** Haili Hu, Qingqing Ma, Yan Wang, Wangsheng Song, Hongyu Xu

**Affiliations:** 1 Anhui Women and Children’s Medical Center, Hefei, 230001 Anhui China; 2 Maternal and Child Medical Center, Anhui Medical University, Hefei, 230001 China; 3 Hefei Women and Children Health Center, Hefei, 230092 China

**Keywords:** Primary carnitine deficiency, Newborn screening, Tandem mass spectrometry, *SLC22A5* gene mutation

## Abstract

**Background:**

Primary carnitine deficiency (PCD) is a rare autosomal recessive fatty acid oxidation disorder caused by variants in the *SLC22A5* gene, with its prevalence and the spectrum of mutations in *SLC22A5* varying across races and regions. This study aimed to analyze the clinical and genetic characteristics of PCD patients, including newborns and their mothers, identified by newborn screening (NBS) in Hefei, China.

**Methods:**

The dried blood spot samples from newborns were analyzed using tandem mass spectrometry (MS/MS) from July 2015 to December 2024. Newborns and their mothers with low free carnitine (C0) levels identified during initial screening were subsequently recalled. Next-generation sequencing was employed to analyze gene mutations in patients whose rescreening results indicated that C0 levels remained below the critical reference value.

**Results:**

A total of 897,050 newborns were screened for PCD, and 46 cases were diagnosed, resulting in an incidence rate of 1 in 19,501. Among the screened population, 34 mothers were identified as PCD patients. A total of 26 different variants were detected in the *SLC22A5* gene, including four novel variants found in both PCD newborns and their mothers (c.253 C > T, c.976_977delinsAGCAGT, c.384dup, and c.236_271del). Of the 44 PCD newborns tested at our center, seven exhibited homozygous mutations, 35 exhibited compound heterozygous mutations, and two cases showed no detectable gene mutation. The most common mutation was c.1400 C > G (45.88%), followed by c.51 C > G (16.47%) and c.760 C > T (8.24%). Among the 34 PCD mothers, 15 had homozygous mutations and 19 had compound heterozygous mutations; 60.29% of the mutations were c.1400 C > G. The C0 levels in patients with *SLC22A5* truncation mutations were significantly lower than those in the non-truncation mutation group (*P* < 0.05). Furthermore, within the truncation mutation group, the C0 levels of patients with the S467C mutation were higher than those of patients without the S467C mutation (*P* < 0.05).

**Conclusions:**

MS/MS combined with genetic testing could effectively enhance the diagnostic accuracy of PCD. Our study identified four novel mutations, expanding the variant spectrum of the *SLC22A5* gene.

## Introduction

Primary carnitine deficiency (PCD) is an autosomal recessive disorder of fatty acid oxidation caused by mutations in the *SLC22A5* gene, which encodes the organic cation transporter 2 (OCTN2) [1]. PCD causes increased urinary carnitine excretion, which leads to low plasma and intracellular carnitine levels. The clinical manifestations of PCD vary widely, encompassing a spectrum from asymptomatic presentations to acute metabolic decompensation in early childhood, as well as progressive hypertrophic cardiomyopathy, myopathy, and encephalopathy in adult [2, 3]. Untreated patients with PCD may experience sudden death [4], emphasizing the importance of early diagnosis and treatment to prevent metabolic decompensation and ensure favorable long-term outcomes. Due to the severity of symptoms and the availability of effective treatment, PCD has been included in newborn screening (NBS) panels in a number of countries [5, 6]. Early diagnosis through NBS and timely supplementation of L-carnitine during the neonatal period, can effectively prevent adverse outcomes [7]. However, the inclusion of PCD in NBS panels facilitated the identification of mothers with PCD, but most of these mothers were asymptomatic at the time of diagnosis and had no prior symptoms that could be associated with PCD [2, 8]. Due to the incomplete understanding of the benefits of NBS, along with the potential harms associated with the identification and treatment of asymptomatic individuals, New Zealand has ceased screening for PCD through NBS [9].

NBS for PCD is widely conducted in China by measuring free carnitine (C0) levels in dried blood spot (DBS) samples using tandem mass spectrometry (MS/MS). However, there are significant regional differences in the incidence of PCD within China, ranging from 1 in 31,000 to 1 in 2,100 [10–12]. Several studies have identified the variants c.760 C > T (p.R254*), c.51 C > G (p.F17L), and c.1400 C > G (p.S467C) as the three most common genetic variants in the Chinese population [13, 14]. But the frequency of the most prevalent variant varies across different regions. The present study was conducted to analyze the clinical, biochemical, and molecular genetic characteristics of PCD patients identified through NBS in Hefei, aiming to provide valuable insights for large sample screening and understanding the variant spectrum of the *SLC22A5* gene.

## Materials and methods

### Study population

A total of 897,050 newborns delivered in Hefei medical midwifery institutions were included in the present study from July 2015 to December 2024. Written informed consent was obtained from the parents or guardians of all newborns. Our study was approved by the Ethics Committee of Anhui Women and Children’s Medical Center (Approval No: 2023-011), in accordance with the ethical standards of the Helsinki declaration and its later amendments or comparable ethical standards.

### Newborn screening for PCD

Three drops of heel blood were collected from newborns who were less than 72 h old and fully breastfed. The blood samples were placed onto specialized filter paper and allowed to air dry naturally. Within five working days, the DBS samples were transported under cold-chain conditions to the laboratory of Hefei Neonatal Disease Screening Center for testing. DBS samples were analyzed by MS/MS (Waters TQD, United States). The acylcarnitine profile were determined using underivatized kits produced by PE Corporation (Finland) in strict accordance with standard laboratory operating procedures. The cut-off value of C0 was 10–55 µmol/L. Newborns with C0 < 10 µmol/L and their mothers were recalled for MS/MS detection again after initial screening, and genetic mutations analysis in *SLC22A5* was performed if C0 test results were still low.

### Genetic analysis

The blood DNA isolation kit was used to extract genomic DNA from the DBS samples of newborns who were suspected to be positive by MS/MS and the peripheral blood of their parents. Then the exons and ~ 20 bp flanking intron sequences of 86 genes including *SLC22A5* gene were amplified by polymerase chain reaction (PCR) for next-generation sequencing (NGS) and analysis. The suspected variants of *SLC22A5* gene detected in the newborns and their parents were verified by Sanger sequencing. The normal human sequence was used as a reference to determine mutations, and the gene sequencing results were compared with the data in HGMD, LOVD, Clin Var and dbSNP databases to obtain the pathogenic mutation site information. All variants were interpreted according to the American College of Medical Genetics and Genomics (ACMG) guidelines (2015).

### Diagnosis of PCD

Diagnosis of PCD was made based on the C0 levels, genetic variants, and clinical symptoms of patients. PCD was identified by C0 < 10 µmol/L and the presence of two variants in *SLC22A5*. If only one or no *SLC22A5* gene mutation was detected, C0 should be tested again under the condition of full feeding. If C0 remains below 10 µmol/L in three consecutive tests and secondary causes such as maternal origin or nutritional deficiencies are excluded, a diagnosis of PCD can also be established [12]. If the mother exhibits a decrease in C0 levels and is otherwise healthy and non-vegetarian, a diagnosis of PCD in the mother can be confirmed through genetic testing. If the infant also demonstrates a decrease in C0 levels, it suggests that the infant has maternal carnitine deficiency [15].

### Treatment and follow-up

Once a diagnosis of PCD is confirmed, immediate treatment with L-carnitine at a dosage of 50–200 mg/kg/day is recommended. The medication should be administered orally in divided doses until the C0 level returns to normal, after which a maintenance dose should be given. It is advised that mothers of PCD patients also undergo L-carnitine treatment. Infants should continue to be breastfed, and if their blood C0 levels remain low, they should also receive L-carnitine treatment [5, 16]. If the infant is not breastfed and is receiving formula milk, there is sufficient L-carnitine in the formula, and if the infant’s C0 level is normal, additional L-carnitine supplementation is not necessary.

### Statistical analysis

SPSS 26.0 (SPSS Inc., Chicago, IL, United States) was used for statistical analysis. Data following a normal distribution were presented as mean ± standard deviation. The t-test was used to compare differences between PCD and non-PCD groups, and a level of *P* < 0.05 was considered statistically significant (α = 0.05).

## Results

### NBS for PCD

From July 1, 2015 to December 31, 2024, a total of 897,050 newborns were screened by MS/MS in Hefei Neonatal Disease Screening Center, 3,151 cases were detected with C0 < 10 µmol/L, and the positive rate was 0.35%. Among them, 3,099 newborns were successfully recalled, and the positive recall rate was 98.35% (3,099/3,151). Finally, 46 newborns were diagnosed with PCD, including 27 males and 19 females. In summary, the incidence of PCD in Hefei was about 1/19,501. In addition, 34 mothers were diagnosed with PCD, the detection rate was 1/26,384. Among them, a mother and a son were both diagnosed with PCD.

### Clinical and biochemical characteristics of PCD patients

Among the 46 PCD newborns, 34 cases were followed up in our center and L-carnitine supplementation was provided immediately for PCD patients after diagnosis. After treatment, the C0 levels of these 34 patients were maintained within the range of 20–30 µmol/L (Table 1). Among the 34 children, the follow-up period ranged from 1 to 106 months, during which liver function, myocardial enzymes, blood glucose, electrocardiograms, and cardiac and hepatic ultrasound were monitored in 23 of the participants. Fifteen children exhibited elevated CK and/or CKMB levels, and two cases suggested possible right ventricular hypertrophy on electrocardiogram. Other findings included three instances of sinus tachycardia, one case of incomplete bundle branch block, and one case of sinus arrhythmia. Ultrasound revealed one child with an uneven echo in the left liver lobe and another with mild mitral regurgitation on echocardiogram. Importantly, none of the children displayed clinical symptoms, and their physical, motor, and language developmental assessments were normal. By increasing the dosage of L-carnitine, improving feeding practices, and adjusting dietary structure, the creatine kinase isoenzymes returned to normal levels in the aforementioned 15 cases.

**Table 1:** Genetic test results of PCD newborns

Case	Gender	C0 levels, µmol/L				SLC22A5 gene mutation			
		Initial screening	Rescreening	After treatment		Allele 1		Allele 2	
						Variants DNA (Protein)	Locations	Variants DNA (Protein)	Locations
1	Male	8.90	6.06	60.69		c.1400 C > G (p.S467C)	Exon 8	c.1400 C > G (p.S467C)	Exon 8
2	Male	8.16	5.92	29.47		c.1400 C > G (p.S467C)	Exon 8	c.1400 C > G (p.S467C)	Exon 8
3	Female	8.75	7.60	33.59		c.1400 C > G (p.S467C)	Exon 8	c.1400 C > G (p.S467C)	Exon 8
4	Female	5.53	7.47	43.87		c.1400 C > G (p.S467C)	Exon 8	c.1400 C > G (p.S467C)	Exon 8
5	Male	8.05	5.26	38.83		c.1400 C > G (p.S467C)	Exon 8	c.1400 C > G (p.S467C)	Exon 8
6	Male	6.29	6.33	34.31		c.1400 C > G (p.S467C)	Exon 8	c.1400 C > G (p.S467C)	Exon 8
7	Female	8.77	8.66	89.62		c.1400 C > G (p.S467C)	Exon 8	c.1400 C > G (p.S467C)	Exon 8
8	Female	6.74	5.06	22.85		c.51 C > G (p.F17L)	Exon 1	c.1400 C > G (p.S467C)	Exon 8
9	Male	4.87	7.75	46.84		c.51 C > G (p.F17L)	Exon 1	c.1400 C > G (p.S467C)	Exon 8
10	Male	5.27	4.88	41.55		c.51 C > G (p.F17L)	Exon 1	c.1400 C > G (p.S467C)	Exon 8
11	Female	3.53	4.23	29.86		c.51 C > G (p.F17L)	Exon 1	c.1400 C > G (p.S467C)	Exon 8
12	Female	7.85	3.99	26.54		c.51 C > G (p.F17L)	Exon 1	c.1400 C > G (p.S467C)	Exon 8
13	Male	9.69	4.37	19.68		c.51 C > G (p.F17L)	Exon 1	c.1400 C > G (p.S467C)	Exon 8
14	Male	6.22	5.37	NA		c.51 C > G (p.F17L)	Exon 1	c.1400 C > G (p.S467C)	Exon 8
15	Male	6.01	4.01	NA		c.51 C > G (p.F17L)	Exon 1	c.1400 C > G (p.S467C)	Exon 8
16	Female	5.34	4.15	NA		c.51 C > G (p.F17L)	Exon 1	c.1400 C > G (p.S467C)	Exon 8
17	Female	7.81	4.80	38.09		c.51 C > G (p.F17L)	Exon 1	c.1400 C > G (p.S467C)	Exon 8
18	Male	4.26	3.39	9.69		c.845G > A (p.R282Q)	Exon 5	c.976_977delinsAGCAGT (p.Q326Sfs*40)^#^	Exon 6
19	Female	7.16	5.29	NA		c.428 C > T (p.P143L)	Exon 2	c.1400 C > G (p.S467C)	Exon 8
20	Male	6.20	6.09	NA		c.428 C > T (p.P143L)	Exon 2	c.1400 C > G (p.S467C)	Exon 8
21	Female	5.25	3.08	17.49		c.428 C > T (p.P143L)	Exon 2	c.760 C > T (p.R254*)	Exon 4
22	Male	5.86	2.87	14.01		c.505 C > T (p.R169W)	Exon 3	c.1400 C > G (p.S467C)	Exon 8
23	Male	5.28	5.89	NA		c.797 C > T (p.P266L)	Exon 4	c.1400 C > G (p.S467C)	Exon 8
24	Female	8.64	5.35	NA		c.338G > A (p.C113Y)	Exon 1	c.1400 C > G (p.S467C)	Exon 8
25	Male	8.4	5.23	22.4		c.1252 C > T (p.Q418*)	Exon 7	c.1400 C > G (p.S467C)	Exon 8
26	Male	2.9	2.14	20.31		c.51 C > G (p.F17L)	Exon 1	c.760 C > T (p.R254*)	Exon 4
27	Male	5.49	4.49	39.89		c.760 C > T (p.R254*)	Exon 4	c.1400 C > G (p.S467C)	Exon 8
28	Female	4	4.37	28.18		c.760 C > T (p.R254*)	Exon 4	c.1400 C > G (p.S467C)	Exon 8
29	Female	6.56	5.74	NA		c.760 C > T (p.R254*)	Exon 4	c.1400 C > G (p.S467C)	Exon 8
30	Female	6.17	5.39	25.14		c.760 C > T (p.R254*)	Exon 4	c.1400 C > G (p.S467C)	Exon 8
31	Female	6.32	4.06	32.7		c.865 C > T (p.R289*)	Exon 5	c.1400 C > G (p.S467C)	Exon 8
32	Male	5.23	3.87	12.76		c.865 C > T (p.R289*)	Exon 5	c.1400 C > G (p.S467C)	Exon 8
33	Male	3.06	2.99	10.24		c.865 C > T (p.R289*)	Exon 5	c.1196G > A (p.R399Q)	Exon 7
34	Male	5.73	6.46	19.77		c.679 C > A (p.R227S)	Exon 4	c.1093 A > C (p.T365P)	Exon 7
35	Female	5.19	4.78	13.15		c.1093 A > C (p.T365P)	Exon 7	c.1400 C > G (p.S467C)	Exon 8
36	Female	6.92	8.65	21.52		c.797 C > T (p.P266L)	Exon 4	c.1195 C > T (p.R399W)	Exon 7
37	Female	5.98	3.98	18.44		c.253 C > T (p.R85W) ^#^	Exon 1	c.1400 C > G (p.S467C)	Exon 8
38	Female	4.26	4.22	12.7		c.760 C > T (p.R254*)	Exon 4	c.1195 C > T (p.R399W)	Exon 7
39	Male	7.7	3.54	24.19		c.1362T > G (p.Y454*)	Exon 8	c.1400 C > G (p.S467C)	Exon 8
40	Male	8.98	8.67	23.67		c.51 C > G (p.F17L)	Exon 1	c.694 A > G (p.T232A)	Exon 4
41	Male	5.33	9.05	24.91		c.51 C > G (p.F17L)	Exon 1	c.1520T > C (p.L507S)	Exon 9
42	Male	4.38	4.07	NA		c.51 C > G (p.F17L)	Exon 1	c.572 A > G (p.K191R)	Exon 3
								c.1229G > A (p.G410D)	Exon 7
43	Male	3.63	2.78	14.05		No SLC22A5 gene mutation was detected			
44	Female	9.03	9.02	NA		No SLC22A5 gene mutation was detected			
45	Male	9.57	7.61	NA		NA	NA	NA	NA
46	Male	7.03	6.85	NA		NA	NA	NA	NA

F, female; M, male; #, novel mutation; NA, not available

Among the 34 PCD mothers, all reported no significant abnormalities in cardiac and liver function during physical examinations, although 2 of them complained of easy fatigue. Out of the 13 PCD mothers that received treatment with L-carnitine, only 8 cases achieved adequate adherence, resulting in C0 levels reaching normal range (Table 2). Except for 9 cases lost to follow-up, the C0 levels of 25 newborns born to the PCD mothers returned to normal when the mothers received L-carnitine treatment or when formula was added to their feeding regimen.

**Table 2:** Genetic test results of PCD mothers

Case	Age	C0 levels, µmol/L			SLC22A5 gene mutation			
		Initial screening	After treatment		Allele 1		Allele 2	
					Variants DNA (Protein)	Locations	Variants DNA (Protein)	Locations
1	28	1.59	NA		c.236_271del (p.79_91del)^#^	Exon 1	c.1400 C > G (p.S467C)	Exon 8
2	27	3.38	NA		c.51 C > G (p.F17L)	Exon 1	c.1400 C > G (p.S467C)	Exon 8
3	29	1.69	NA		c.51 C > G (p.F17L)	Exon 1	c.1400 C > G (p.S467C)	Exon 8
4	29	1.51	NA		c.51 C > G (p.F17L)	Exon 1	c.1400 C > G (p.S467C)	Exon 8
5	35	1.31	15.13		c.51 C > G (p.F17L)	Exon 1	c.1400 C > G (p.S467C)	Exon 8
6	29	2.40	NA		c.51 C > G (p.F17L)	Exon 1	c.1400 C > G (p.S467C)	Exon 8
7	27	3.08	NA		c.797 C > T (p.P266L)	Exon 4	c.1400 C > G (p.S467C)	Exon 8
8	30	6.39	NA		c.1400 C > G (p.S467C)	Exon 8	c.1400 C > G (p.S467C)	Exon 8
9	29	3.07	NA		c.1400 C > G (p.S467C)	Exon 8	c.1400 C > G (p.S467C)	Exon 8
10	22	2.46	NA		c.1400 C > G (p.S467C)	Exon 8	c.1400 C > G (p.S467C)	Exon 8
11	35	2.83	13.75		c.1400 C > G (p.S467C)	Exon 8	c.1400 C > G (p.S467C)	Exon 8
12	22	2.7	NA		c.1400 C > G (p.S467C)	Exon 8	c.1400 C > G (p.S467C)	Exon 8
13	27	1.98	12.92		c.1400 C > G (p.S467C)	Exon 8	c.1400 C > G (p.S467C)	Exon 8
14	28	2.45	18.21		c.1400 C > G (p.S467C)	Exon 8	c.1400 C > G (p.S467C)	Exon 8
15	36	2.55	NA		c.1400 C > G (p.S467C)	Exon 8	c.1400 C > G (p.S467C)	Exon 8
16	33	2.23	20.93		c.1400 C > G (p.S467C)	Exon 8	c.1400 C > G (p.S467C)	Exon 8
17	28	2.04	NA		c.1400 C > G (p.S467C)	Exon 8	c.1400 C > G (p.S467C)	Exon 8
18	29	2.61	NA		c.1400 C > G (p.S467C)	Exon 8	c.1400 C > G (p.S467C)	Exon 8
19	30	2.72	8.25		c.1400 C > G (p.S467C)	Exon 8	c.1400 C > G (p.S467C)	Exon 8
20	25	2.45	NA		c.1400 C > G (p.S467C)	Exon 8	c.1400 C > G (p.S467C)	Exon 8
21	37	1.04	NA		c.760 C > T (p.R254*)	Exon 4	c.1195 C > T (p.R399W)	Exon 7
22	24	4.67	18.5		c.51 C > G (p.F17L)	Exon 1	c.1198 C > T (p.R400C)	Exon 7
23	26	0.9	8.55		c.865 C > T (p.R289*)	Exon 5	c.1400 C > G (p.S467C)	Exon 8
24	21	2.07	NA		c.384dup (p.V129Cfs*9)^#^	Exon 1	c.1400 C > G (p.S467C)	Exon 8
25	29	1.11	12.95		c.760 C > T (p.R254*)	Exon 4	c.1400 C > G (p.S467C)	Exon 8
26	28	1.56	6.06		c.760 C > T (p.R254*)	Exon 4	c.1400 C > G (p.S467C)	Exon 8
27	30	1.04	16.12		c.760 C > T (p.R254*)	Exon 4	c.1400 C > G (p.S467C)	Exon 8
28	32	1.72	7.21		c.338G > A (p.C113Y)	Exon 1	c.1400 C > G (p.S467C)	Exon 8
29	35	2.81	NA		c.1052 + 3 A> G	Intron6	c.1400 C > G (p.S467C)	Exon 8
30	33	1.43	NA		c.1252 C > T (p.Q418*)	Exon 7	c.1400 C > G (p.S467C)	Exon 8
31	35	2.07	NA		c.51 C > G (p.F17L)	Exon 1	c.428 C > T (p.P143L)	Exon 2
32	33	2.08	6.71		c.338G > A (p.C113Y)	Exon 1	c.428 C > T (p.P143L)	Exon 2
33	30	3.61	NA		c.832 C > T (p.P278S)	Exon 5	c.832 C > T (p.P278S)	Exon 5
34	25	3.03	NA		c.428 C > T (p.P143L)	Exon 2	c.428 C > T (p.P143L)	Exon 2

#, novel mutation; NA, not available

### Molecular genetic analysis

**Fig. 1:**
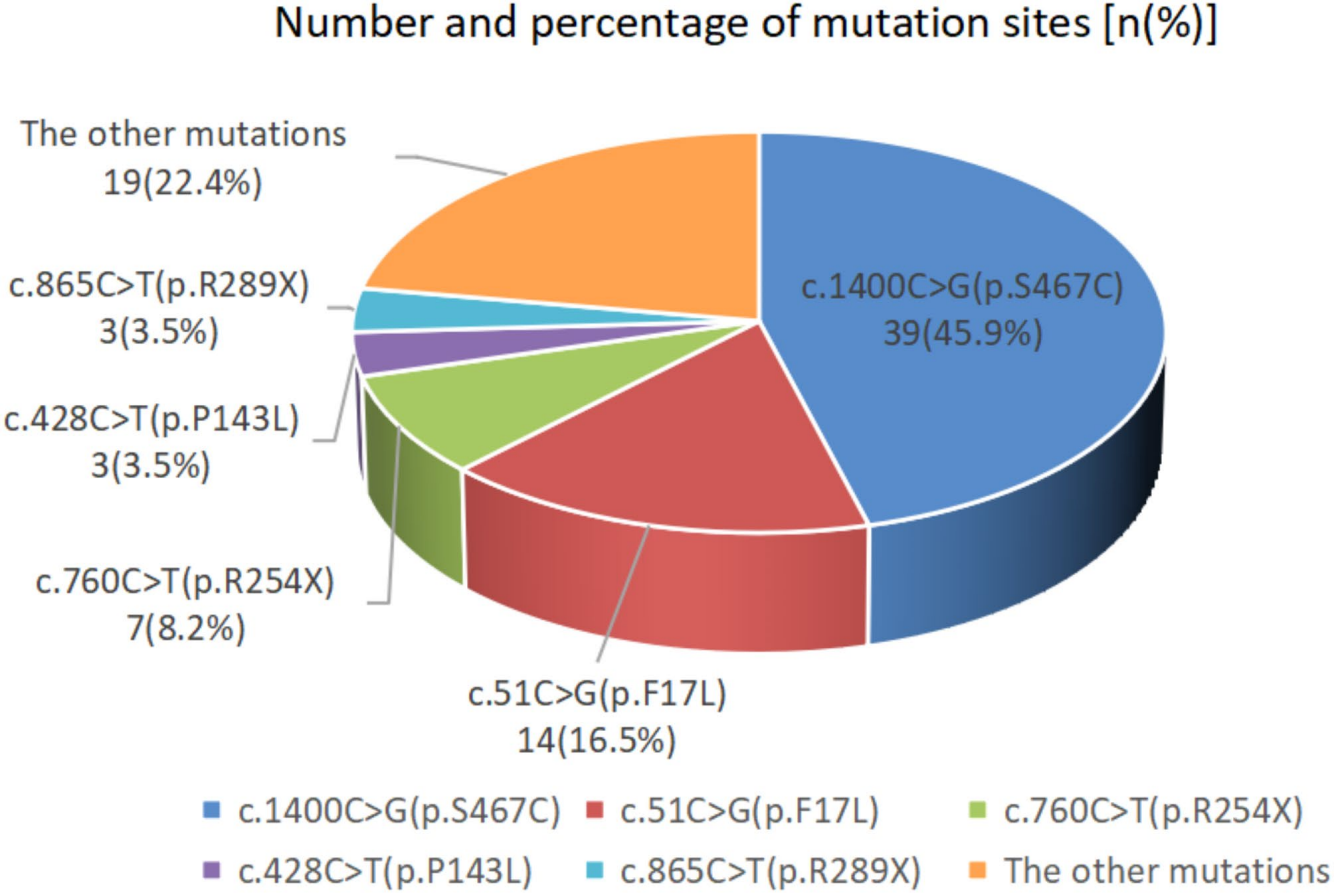
Number and percentage of mutation sites in the *SLC22A5* gene

Of the 46 newborns diagnosed with PCD, 44 were confirmed by genetic testing at our center, and two newborns were diagnosed with PCD at other hospitals and no mutation data was collected. Among 44 newborns, 7 were identified with homozygous mutations, 35 newborns had compound heterozygous mutations, and no mutation in *SLC22A5* gene was detected in two newborns, but under the condition of full feeding and ruling out secondary carnitine deficiency, C0 < 10 µmol/L was detected for 3 consecutive times, accompanied by various acylcarnitine reductions, and finally diagnosed with PCD. A total of 85 mutations were identified, including 1 newborn with 3 mutations. These mutations were classified into 21 different types, including 16 missense mutations, 4 nonsense mutations, and 1 frameshift mutation, distributed in exons 1–9 of *SLC22A5* gene, and the majority of these mutations were concentrated in exon 1, 4, and 8, accounting for 78.82% (67/85) of all mutations. Among the 21 types of mutations, the most common was the c.1400 C > G (p.S467C), accounting for 45.88% (39/85) of cases. The second most common was the c.51 C > G (p.F17L), accounting for 16.47% (14/85), followed by the c.760 C > T (p.R254*), which accounted for 8.24% (7/85) (Fig. 1). Additionally, two novel mutations were identified: (1) c.253 C > T (p.R85W) was classified as a variant of uncertain significance (PM2_P + PM3 + PP4), and (2) c.976_977delinsAGCAGT (p.Q326Sfs*40) was classified as a pathogenic variant (PVS1 + PM2_P + PM3_P) according to the ACMG guidelines. The genetic characteristics of all PCD newborns are provided in Table 1.

In 34 cases of PCD mothers, 15 cases were identified with homozygous mutations and 19 cases with compound heterozygous mutations. A total of 68 mutations were detected, consisting of 14 different types, including 8 missense mutations, 3 nonsense mutations, 1 frameshift mutation, 1 deletion mutation and 1 splicing mutation. These mutations were distributed across exons 1, 2, 4, 5, 7, and 8 (as well as intron 6) of the *SLC22A5* gene, with the majority concentrated in exons 1 and 8, accounting for 76.47% of all mutation sites (52/68). Among the 14 types of mutations, the most common was the c.1400 C > G (p.S467C), accounting for 60.29% (41/68); followed by c.51 C > G (p.F17L), accounting for 10.29% (7/68); c.760 C > T (p.R254*) and c.428 C > T (p.P143L) each accounted for 5.88% (4/68). Two novel mutations were identified. According to the ACMG guidelines, the c.236_271del (p.79_91del) variant was classified as a variant of uncertain significance (PM2_P + PM4), while c.384dup (p.V129Cfs*9) was classified as a pathogenic variant (PVS1 + PM2 + PP4). The genetic characteristics of PCD mothers are provided in Table 2.

### Relationship between genotype and biochemical indicators

PCD newborns exhibited four kinds of nonsense mutations on the *SLC22A5* gene, namely c.760 C > T (p.R254*), c.865 C > T (p.R289*), c.1252 C > T (p.Q418*), and c.1362T > G (p.Y454*), as well as one frameshift mutation, c.976_977delinsAGCAGT (p.Q326Sfs*40). PCD mothers had three nonsense mutations, c.760 C > T (p.R254*), c.865 C > T (p.R289*) and c.1252 C > T (p.Q418*), as well as one frameshift mutation, c.384dup (p.V129Cfs*9). All these mutations can result in truncation proteins. Based on whether the mutations generate truncation proteins, PCD newborns and PCD mothers were divided into the truncation mutation group and non-truncation mutation group, respectively. The truncation mutation group of PCD newborns exhibited lower C0 levels compared to the non-truncation mutation group in initial screening and rescreening, with statistically significant differences (*P* < 0.05). Similarly, the truncation mutation group of PCD mothers showed lower levels of C0 compared to the non-truncation mutation group, with statistically significant differences (*P* < 0.05). Refer to Table 3 for detailed information. In addition, among the PCD newborns with *SLC22A5* truncation mutation, the group with S467C mutation had higher C0 level in initial and rescreening than the group without S467C mutation, and the difference was statistically significant (*P* < 0.05), see Table 4 for details.

**Table 3:** Comparison of C0 level between the Truncation mutation group and non-truncation mutation group

Group	PCD newborns				PCD mothers	
	No.	C0 in initial screening (µmol/L)	C0 in rescreening (µmol/L)		No.	C0 level (µmol/L)
Truncation mutation group	13	5.35 ± 1.67	4.04 ± 1.03		7	1.31 ± 0.41
Non-truncation mutation group	29	6.67 ± 1.60	5.76 ± 1.68		27	2.65 ± 1.04
*t*		2.435	3.409			3.317
*P*		0.019	0.002			0.002

**Table 4:** Comparison of C0 level between the Truncation mutation group concurrently with and without S467C mutation

Group	No.	C0 in initial screening (µmol/L)	C0 in rescreening (µmol/L)
Truncation mutation concurrently with S467C mutation	8	6.23 ± 1.39	4.59 ± 0.79
Truncation mutation concurrently without S467C mutation	5	3.95 ± 0.97	3.16 ± 0.75
*t*		3.201	3.225
*P*		0.008	0.008

## Discussion

Our study analyzed the results of a nine-year screening for primary carnitine deficiency (PCD) in Hefei City, revealing a relatively high incidence of PCD in the region. It was found that the incidence of maternally inherited PCD, due to mothers being PCD patients, is higher compared to other regions in China. The present study identified c.1400 C > G (p.S467C), c.51 C > G (p.F17L), and c.760 C > T (p.R254X) as hotspot mutations of the *SLC22A5* gene in this area. Additionally, four novel mutations—c.236_271del (p.79_91del), c.253 C > T (p.R85W), c.384dup (p.V129CfsX9), and c.976_977delinsAGCAGT (p.Q326SfsX40)—were discovered, enriching the mutation spectrum of the *SLC22A5* gene. The study found that children carrying truncating mutations had lower levels of C0, suggesting that the activity of their target protein, OCTN2, is reduced. In contrast, the c.1400 C > G (p.S467C) mutation appears to have a lesser impact on transport activity compared to other types of gene mutations, allowing for a relatively higher residual activity of the carnitine transporter.

PCD is a potentially life-threatening disease. Due to the significant improvement in prognosis when diagnosed and treated early, many countries and regions have included it in newborn disease screening programs [14]. Previous studies have shown significant ethnic variations in the incidence of this disease, with the highest incidence of 1/300 in the Faroe Islands, reported incidence of 1/20,000–1/70,000 in the United States, 1/40,000 in Japan, and 1/120,000 in Australia [17]. The incidence of PCD reported in different regions of China is different. In Taiwan, the incidence was reported as 1/70,000 [18], while Xinhua Hospital Affiliated to Shanghai Jiao Tong University School of Medicine reported an incidence of 1/ 31,200 [12], Children’s Hospital of Zhejiang University School of Medicine reported an incidence of 1/ 30,182 [13], the reported incidence in Jiangsu Province was 1/29,778 [19], the incidence rates reported in Quanzhou was 1/11,189 [14]. From July 1, 2015 to December 31, 2024, a total of 897,050 newborns were screened in Hefei, and 46 cases were diagnosed with PCD, with an incidence rate of 1/19,501, which was lower than that of Quanzhou. Furthermore, 34 mothers were diagnosed with PCD, the detection rate was 1/26,384, which was higher than the 1/54,137 reported by Children’s Hospital of Zhejiang University School of Medicine [13].

The *SLC22A5* gene is located on chromosome 5q31.1 and consists of 10 exons and 9 introns spanning approximately 30 kb. OCTN2 protein encoded by *SLC22A5* is a transmembrane protein composed of 557 amino acids, including 12 transmembrane domains and an ATP binding site. Reported mutation sites involve exons 1–9 and introns 3, 7, and 8 [20]. To date, more than 180 types of mutations have been identified, with missense mutations being the most common, followed by nonsense mutations, frameshift mutations, and splice site mutations [7]. The variants of *SLC22A5* gene reported in different regions are different. In a population study in Texas U. S. A., the most common mutations were c.424G > T (p.A142S) and c.136 C > T (p.P46S) [21]. In Italy, c.505 C > T (p.R169W) was the most frequently observed mutation, while in Japan, c.1400 C > G (p.S467C) and c.396G > A (p.W132*) were the most common mutations [22]. In China, the most common mutation reported in Quanzhou in 2021 was c.760 C > T (p.R254*), which was consistent with the findings in Taiwan [14]. In 2022, a study in Shanghai, reported that the most common mutation was c.51 C > G (p.F17L) [12]. In our study, the most common mutations in newborn PCD patients in Hefei were c.1400 C > G (p.S467C), c.51 C > G (p.F17L), and c.760 C > T (p.R254*), which were consistent with the common mutations reported in Zhejiang [13], and the mutation frequencies were 45.88% (39/85), 16.47% (14/85), and 8.24% (7/85), respectively.

In the present study, two patients did not display detectable mutation sites; however, with adequate nutritional intake and the exclusion of secondary carnitine deficiency, they exhibited three consecutive measurements below 10 µmol/L, accompanied by reductions in various acylcarnitines, leading to a clinical diagnosis of PCD. The absence of detectable mutations in these patients may be due to the limitations of current high-throughput sequencing techniques, which primarily target exonic regions and the surrounding 20 base pairs of intronic sequences, potentially overlooking deep intronic and promoter region variations. Furthermore, methylation modifications of the *SLC22A5* gene may contribute to this phenomenon. Certain genes may appear normal at the sequence level yet could be functionally impaired due to epigenetic mechanisms. Therefore, relying exclusively on genetic testing results to ascertain whether a child has PCD is inadequate, a comprehensive assessment must also include the patient’s biochemical markers.

Case 42 in Table 1 and Case 5 in Table 2 refer to a mother and her newborn. In Case 42, as presented in Table 1, three mutation sites in the *SLC22A5* gene were identified: one inherited from the mother and two from the father. The mother exhibited two mutation sites in the *SLC22A5* gene, indicating that both mutations were inherited from her parents. Both the mother and child demonstrated C0 values of less than 10 µmol/L, along with multiple reductions in acylcarnitines, resulting in a diagnosis of PCD. The identification of a mother as a patient with PCD should not be the sole basis for determining whether her newborn is a carrier of the condition. If a newborn is formula-fed and does not show a significant increase in C0 levels, genetic testing should still be considered to determine the possibility of the newborn being a PCD patient. The best approach is to recommend simultaneous genetic testing for both the mother and newborn when the C0 levels are low. Combining MS/MS screening with genetic testing is an effective approach to improve the efficiency of newborn disease screening [14].

The common mutation sites in PCD mothers are essentially the same as those in PCD newborns, but with different proportions. The c.1400 C > G mutation accounts for 60.29% (41/68), c.51 C > G accounts for 10.29% (7/68), and c.760 C > T accounts for 5.88% (4/68). The c.1400 C > G mutation is located in exon 8, resulting in the substitution of serine with cysteine at codon 467. This mutation is positioned in the 11th transmembrane domain of the OCTN2 protein, affecting the binding of carnitine to OCTN2, thereby reducing carnitine transport capacity. Studies have shown that this mutation often results in asymptomatic clinical outcomes, suggesting a milder degree of disease manifestation caused by this mutation [15]. Among 34 PCD mothers, 13 of them were homozygous for the c.1400 C > G mutation, while the remaining 15 were carriers of this mutation. The c.1400 C > G mutation had a relatively high frequency, suggesting a potential association with milder disease manifestations, allowing affected females to have normal pregnancies and deliveries. On the other hand, the c.760 C > T mutation is a severe mutation that leads to premature termination of protein synthesis, resulting in extremely low residual activity of the OCTN2 protein [23]. Some researchers propose that the lower detection rate of the c.760 C > T mutation in PCD mothers may be related to the increased risk of premature death in untreated patients who are homozygous for this mutation [13].Furthermore, we identified four novel mutations, including c.236_271del (p.79_91del) and c.253 C > T (p.R85W) were of unknown clinical significance, while c.384dup (p.V129Cfs*9) and c.976_977delinsAGCAGT (p.Q326Sfs*40) were classified as pathogenic variants, thus enriching the mutation profile of the *SLC22A5* gene.

Among all the PCD patients in this study, four types of nonsense mutations were detected: c.760 C > T (p.R254*), c.865 C > T (p.R289*), c.1252 C > T (p.Q418*), and c.1362T > G (p.Y454*), additionally, two frameshift mutations were identified: c.976_977delinsAGCAGT (p.Q326Sfs*40) and c.384dup (p.V129Cfs*9). All of these mutations are pathogenic variants that result in premature termination of protein synthesis, leading to the formation of truncation proteins with no or abnormal functionality. Consequently, OCTN2 protein function is impaired, affecting carnitine transport [24]. The analysis of C0 levels in PCD newborns and their mothers revealed that patients with truncation mutations exhibited significantly lower C0 levels compared to those without truncation mutations. This finding aligns with the notion proposed by Rose EC et al. [8] that patients with nonsense or frameshift mutations exhibit extremely weak activity of the target protein OCTN2.

This study also analyzed the difference in C0 levels between two groups of PCD newborns, one group carrying both truncation mutations and the S467C mutation, and the other group without the S467C mutation. We found that the C0 levels of group with the S467C mutation were higher than those without the S467C mutation both in initial and rescreening, and the difference was statistically significant. These findings are consistent with the results of a study by Lin et al. [14], suggesting that the c.1400 C > G (p.S467C) mutation may have a smaller impact on the transporter compared to other types of gene mutations, allowing for a higher OCTN2 protein transport activity to be retained.

The treatment principles for PCD involve avoiding starvation and prolonged high-intensity exercise, as well as lifelong administration of carnitine supplementation therapy to maintain normal plasma free carnitine (C0) levels. Among the 34 children who received follow-up, 15 cases of elevated creatine kinase or creatine kinase isoenzymes exhibited normalization of these levels following an increase in L-carnitine dosage, enhanced nutritional support, and assurance of adequate intake of lean meats, beef, or lamb. Among the 34 PCD mothers, except for two cases reporting fatigue, the others did not exhibit symptoms, similar to the majority of asymptomatic or mildly symptomatic adult patients reported in relevant literature. However, due to the risk of sudden cardiac death, once PCD is diagnosed, standardized treatment, adherence to medication as prescribed, regular follow-up, and avoidance of risk factors are necessary [15]. Studies have shown that PCD mothers are more likely to experience symptoms during pregnancy, possibly due to the increased energy consumption during gestation. Therefore, it is important to closely monitor the levels of plasma free carnitine in pregnant PCD women to avoid energy metabolism disorders resulting from decreased carnitine levels [25]. A multidisciplinary approach and effective communication with the family are essential. Targeted next-generation sequencing (NGS) techniques can guide and support clinicians, ensuring that patients receive the most appropriate clinical management while avoiding unnecessary and/or disproportionate treatments [26–29]. It is crucial to inform PCD patients who are considering reproduction that the efficacy of PCD treatments is well-established and that prenatal diagnosis is available, termination of the pregnancy is not recommended.

## Conclusions

Our study demonstrates that MS/MS combined with genetic testing is an effective approach for PCD diagnosis, and c.1400 C > G (p.S467C), c.51 C > G (p.F17L), and c.760 C > T (p.R254*) are the hotspot mutations of *SLC22A5* gene. Additionally, four new mutations, c.236_271del (p.79_91del), c.253 C > T (p.R85W), c.384dup (p.V129Cfs*9), and c.976_977delinsAGCAGT (p.Q326Sfs*40) have been discovered, thereby enriching the mutation profile of the *SLC22A5* gene. Furthermore, a potential association between the types of gene mutations and patient C0 levels has been observed. As a result, for newborns with low C0 levels detected in initial screening, it is necessary to perform tandem mass spectrometry testing and genetic testing on both the mother and the newborn to differentiate whether it is a maternally derived PCD in the newborn. The effectiveness of L-carnitine therapy for PCD is well-established, and once diagnosed, immediate lifelong administration of L-carnitine supplement therapy is recommended. Given that asymptomatic individuals with PCD may face significant health risks, including fatty liver, cardiomyopathy, and sudden death, it is essential to educate mothers of PCD patients about these potential dangers. A comprehensive evaluation is necessary to determine the appropriateness of medication, along with regular follow-up and monitoring of blood carnitine levels and overall health status.

## Data Availability

The datasets generated and analyzed during the current study are not publicly available due to participant privacy concerns but are available from the corresponding author on reasonable request.

## Abbreviations

DBS: Dried blood spot
NGS: Next-generation sequencing
OCTN2: Organic cation transporter 2
PCD: Primary carnitine deficiency
PCR: Polymerase chain reaction

## References

[1] Cederbaum SD, Koo-McCoy S, Tein I, Hsu BY, Ganguly A, Vilain E, et al. Carnitine membrane transporter deficiency: a long-term follow up and OCTN2 mutation in the first documented case of primary carnitine deficiency. Mol Genet Metab. 2002;77:195–201. 12409266 10.1016/s1096-7192(02)00169-5

[2] Crefcoeur LL, Visser G, Ferdinandusse S, Wijburg FA, Langeveld M, Sjouke B. Clinical characteristics of primary carnitine deficiency: A structured review using a case-by-case approach. J Inherit Metab Dis. 2022;45:386–405. 34997761 10.1002/jimd.12475 PMC9305179

[3] Saudubray JM, Garcia-Cazorla A. Inborn errors of metabolism overview: pathophysiology, manifestations, evaluation, and management. Pediatr Clin North Am. 2018;65:179–208. 29502909 10.1016/j.pcl.2017.11.002

[4] Rasmussen J, Duno M, Lund AM, Steuerwald U, Hansen SH, Joensen HD, et al. Increased risk of sudden death in untreated primary carnitine deficiency. J Inherit Metab Dis. 2020;43:290–6. 31373028 10.1002/jimd.12158

[5] Schimmenti LA, Crombez EA, Schwahn BC, Heese BA, Wood TC, Schroer RJ, et al. Expanded newborn screening identifies maternal primary carnitine deficiency. Mol Genet Metab. 2007;90:441–5. 17126586 10.1016/j.ymgme.2006.10.003

[6] Wilcken B, Wiley V, Sim KG, Carpenter K. Carnitine transporter defect diagnosed by newborn screening with electrospray tandem mass spectrometry. J Pediatr. 2001;138:581–4. 11295726 10.1067/mpd.2001.111813

[7] Longo N, Frigeni M, Pasquali M. Carnitine transport and fatty acid oxidation. Biochim Biophys Acta. 2016;1863:2422–35. 26828774 10.1016/j.bbamcr.2016.01.023 PMC4967041

[8] Rose EC, di San Filippo CA, Ndukwe Erlingsson UC, Ardon O, Pasquali M, Longo N. Genotype-phenotype correlation in primary carnitine deficiency. Hum Mutat. 2012;33:118–23. 21922592 10.1002/humu.21607 PMC3240685

[9] Wilson C, Knoll D, de Hora M, Kyle C, Glamuzina E, Webster D. The decision to discontinue screening for carnitine uptake disorder in new Zealand. J Inherit Metab Dis. 2019;42:86–92. 30740730 10.1002/jimd.12030

[10] Wang X, Fang H. Clinical and gene analysis of fatty acid oxidation disorders found in neonatal tandem mass spectrometry screening. Pharmgenomics Pers Med. 2023;16:577–87. 37305019 10.2147/PGPM.S402760 PMC10254624

[11] Lin Y, Zheng Q, Zheng T, Zheng Z, Lin W, Fu Q. Expanded newborn screening for inherited metabolic disorders and genetic characteristics in a Southern Chinese population. Clin Chim Acta. 2019;494:106–11. 30904546 10.1016/j.cca.2019.03.1622

[12] Chang S, Yang Y, Xu F, Ji W, Zhan X, Gao X, et al. Clinical, biochemical, and molecular genetic characteristics of patients with primary carnitine deficiency identified by newborn screening in Shanghai, China. Front Genet. 2022;13:1062715. 36568374 10.3389/fgene.2022.1062715 PMC9772520

[13] Lin Y, Xu H, Zhou D, Hu Z, Zhang C, Hu L, et al. Screening 3.4 million newborns for primary carnitine deficiency in Zhejiang Province, China. Clin Chim Acta. 2020;507:199–204. 32371215 10.1016/j.cca.2020.04.039

[14] Lin Y, Lin B, Chen Y, Zheng Z, Fu Q, Lin W, et al. Biochemical and genetic characteristics of patients with primary carnitine deficiency identified through newborn screening. Orphanet J Rare Dis. 2021;16:503. 34863234 10.1186/s13023-021-02126-3 PMC8642906

[15] Lee NC, Tang NL, Chien YH, Chen CA, Lin SJ, Chiu PC, et al. Diagnoses of newborns and mothers with carnitine uptake defects through newborn screening. Mol Genet Metab. 2010;100:46–50. 20074989 10.1016/j.ymgme.2009.12.015

[16] El-Hattab AW, Li FY, Shen J, Powell BR, Bawle EV, Adams DJ, et al. Maternal systemic primary carnitine deficiency uncovered by newborn screening: clinical, biochemical, and molecular aspects. Genet Med. 2010;12:19–24. 20027113 10.1097/GIM.0b013e3181c5e6f7

[17] Magoulas PL, El-Hattab AW. Systemic primary carnitine deficiency: an overview of clinical manifestations, diagnosis, and management. Orphanet J Rare Dis. 2012;7:68. 22989098 10.1186/1750-1172-7-68 PMC3495906

[18] Shibata N, Hasegawa Y, Yamada K, Kobayashi H, Purevsuren J, Yang Y, et al. Diversity in the incidence and spectrum of organic acidemias, fatty acid oxidation disorders, and amino acid disorders in Asian countries: selective screening vs. expanded newborn screening. Mol Genet Metab Rep. 2018;16:5–10. 29946514 10.1016/j.ymgmr.2018.05.003 PMC6014585

[19] Yang Y, Wang L, Wang B, Liu S, Yu B, Wang T. Application of Next-Generation sequencing following tandem mass spectrometry to expand newborn screening for inborn errors of metabolism: A multicenter study. Front Genet. 2019;10:86. 30838026 10.3389/fgene.2019.00086 PMC6382741

[20] Li FY, El-Hattab AW, Bawle EV, Boles RG, Schmitt ES, Scaglia F, et al. Molecular spectrum of SLC22A5 (OCTN2) gene mutations detected in 143 subjects evaluated for systemic carnitine deficiency. Hum Mutat. 2010;31:E1632–51. 20574985 10.1002/humu.21311

[21] Chen Y, Lin Q, Zeng Y, Qiu X, Liu G, Zhu W. Gene spectrum and clinical traits of 10 patients with primary carnitine deficiency. Mol Genet Genomic Med. 2021;9:e1583. 33560599 10.1002/mgg3.1583 PMC8077093

[22] Tang NL, Hwu WL, Chan RT, Law LK, Fung LM, Zhang WM. A founder mutation (R254X) of SLC22A5 (OCTN2) in Chinese primary carnitine deficiency patients. Hum Mutat. 2002;20:232. 12204000 10.1002/humu.9053

[23] Frigeni M, Balakrishnan B, Yin X, Calderon FRO, Mao R, Pasquali M, et al. Functional and molecular studies in primary carnitine deficiency. Hum Mutat. 2017;38:1684–99. 28841266 10.1002/humu.23315 PMC5665702

[24] Kilic M, Ozgul RK, Coskun T, Yucel D, Karaca M, Sivri HS, et al. Identification of mutations and evaluation of cardiomyopathy in Turkish patients with primary carnitine deficiency. JIMD Rep. 2012;3:17–23. 23430869 10.1007/8904_2011_36 PMC3509853

[25] Vijay S, Patterson A, Olpin S, Henderson MJ, Clark S, Day C, et al. Carnitine transporter defect: diagnosis in asymptomatic adult women following analysis of acylcarnitines in their newborn infants. J Inherit Metab Dis. 2006;29:627–30. 16865412 10.1007/s10545-006-0376-y

[26] Piccione M, Serra G, Sanfilippo C, Andreucci E, Sani I, Corsello G. A new mutation in EDA gene in X-linked hypohidrotic ectodermal dysplasia associated with keratoconus. Minerva Pediatr. 2012;64:59–64. 22350046

[27] Serra G, Corsello G, Antona V, D’Alessandro MM, Cassata N, Cimador M, et al. Autosomal recessive polycystic kidney disease: case report of a newborn with rare PKHD1 mutation, rapid renal enlargement and early fatal outcome. Ital J Pediatr. 2020;46:154. 33059727 10.1186/s13052-020-00922-4 PMC7560064

[28] Serra G, Giambrone C, Antona V, Cardella F, Carta M, Cimador M, et al. Congenital hypopituitarism and multiple midline defects in a newborn with non-familial Cat eye syndrome. Ital J Pediatr. 2022;48:170. 36076277 10.1186/s13052-022-01365-9 PMC9461219

[29] Piccione M, Serra G, Consiglio V, Di Fiore A, Cavani S, Grasso M, et al. 14q13.1-21.1 deletion encompassing the HPE8 locus in an adolescent with intellectual disability and bilateral microphthalmia, but without holoprosencephaly. Am J Med Genet A. 2012;158A:1427–33. 22581785 10.1002/ajmg.a.35334

